# Chemically Degradable Vitrimers Based on Divanillin Imine Diepoxy Monomer and Aliphatic Diamines for Enhanced Carbon Fiber Composite Applications

**DOI:** 10.3390/polym16192754

**Published:** 2024-09-29

**Authors:** Tommaso Telatin, Silvia De la Flor, Xavier Montané, Àngels Serra

**Affiliations:** 1 Department of Analytical and Organic Chemistry, Universitat Rovira i Virgili, C/Marcel·lí Domingo 1, 43007 Tarragona, Spain; tommaso.telatin@urv.cat; 2 Department of Mechanical Engineering, Universitat Rovira i Virgili, Av. Països Catalans 26, 43007 Tarragona, Spain; silvia.delaflor@urv.cat

**Keywords:** polyimine–epoxy vitrimers, creep resistance, chemical degradation, carbon fiber composites

## Abstract

This study presents the development of a diglycidyl monomer containing two imine groups that can act as dynamic and reversible bonds. During the curing of the monomer with two different amine hardeners, we confirmed the formation of new imine groups due to the transamination reaction between the imine groups of the diepoxy monomer with the amine groups of the hardener. The effect of this structural change was observed in the stress relaxation behavior, resulting in the overlapping of two different relaxation modes. The analytical modelling was able to extract two distinct characteristic relaxation times using a double-element Maxwell model. A second characterization of the stress relaxation process by frequency sweep experiments was performed to corroborate the results obtained, confirming speedy stress relaxation. Acid-catalyzed hydrolysis was performed on the studied materials, demonstrating the complete degradation of the network. We finally confirmed that the synthesized diepoxy compound is suitable for preparing carbon-fiber-reinforced composite materials, demonstrating easy fiber impregnation, fast reshaping, and especially a total degradation of the polymer matrix that allows for the recovery of the carbon fibers in mild conditions. This epoxy–amine system is an excellent candidate for overcoming the traditional limits of thermosets in preparing fiber-reinforced composites.

## 1. Introduction

Since its tremendous impact on our era, the polymer and plastics industry will undeniably play a main role in the essential change of paradigm from a linear to a circular economy. Indeed, on one side, polymer materials are currently finger-pointed as one of the main sources of environmental issues; on the other side, their low cost, lightweight nature, high performance, easy production, and overall durability and adaptability make them the most sustainable and convenient alternative for a broad range of applications [1]. In this class of materials, thermoset polymers undoubtedly represent a fundamental item because of their stability, insolubility, and high mechanical and thermal properties lent by their covalently cross-linked network. The same structural feature that determines their technologically valuable properties is also responsible for their lack of reshapeability, moldability, and recyclability at the end of their useful life [2].

The introduction of reversible bonds in the three-dimensional structure and the formation of covalent adaptable networks (CANs) represent a powerful approach to overcoming the limitations of thermosets [3]. The presence of chemical groups able to undergo equilibrium reactions makes these materials able to rearrange when triggered by a specific stimulus, while they behave as fully covalent networks when the kinetic of the exchange reaction is not boosted [4,5]. The mechanism through which the exchange reaction occurs plays a fundamental role in determining material properties, especially in its transformation processes [6]. Reactions that take place through a dissociative path determine a de-bonding process and, consequently, a drop in the cross-linking density, resulting in a solid-to-liquid transition [7,8].

Conversely, exchange reactions that follow a concerted mechanism maintain the cross-link density unaltered during the exchange process and generate a broader rubbery region than dissociative CANs. The rheological behavior of associative CANs mimics the temperature dependency of vitreous silica exhibiting an Arrhenius relationship between viscosity and temperature [9].

Among all the dynamic chemistries suitable for developing vitrimeric materials, imines represent an attractive option, joining a fast exchange process without needing any catalyst with ease of synthesis and usually cheap building blocks [10]. Imines are obtained from the condensation reaction of aldehydes with primary amine and can undergo acid-catalyzed hydrolysis. Moreover, two different reactions, transamination and imine metathesis, occur by concerted mechanisms and can be exploited as dynamic systems in vitrimers [11,12].

Pure polyimine materials can be obtained by reacting a dialdehyde with a combination of a diamine and a triamine, as deeply investigated by Zhang and coworkers [13,14]. Moreover, the necessity of proceeding through film-casting procedures and the formation of water as a side product during the curing reaction strongly compromise the attraction of these kinds of materials.

Another route involves the preparation of telechelic linear polyimine oligomers as dynamic hardeners. Liang et al. studied a system based on an amine-terminated polyimine oligomer cured with trimethyl citrate [15]. In contrast, Huang and coworkers prepared materials with a similar approach, using polyimine oligomers as a curing agent for a commercial epoxy resin [16]. Our group deeply investigated the effect of the polyimine oligomer length and the proportion of two different amines used in its synthesis in an epoxy–amine system [17]. Despite the promising results of this approach in modulating glass transition temperature and tuning the activation energy for the exchange reaction, this is limited by a complex control of the chemical degradability.

The synthesis of imine-containing epoxy monomers represents a step forward from this point of view, determining a homogeneous distribution of the dynamic groups and, consequently, an easier chemical degradation. Moreover, among thermosets, epoxy resins are one of the most used polymeric resins due to their superior performance in many industrial applications. They can be formulated with a wide selection of curing agents including amines, amides, phenols, carboxylic acids, anhydrides, and thiols, obtaining properties able to cover a broad range of uses [18]. It is evident, therefore, how the development of epoxy compounds that combine the excellent properties of traditional epoxy resins with a vitrimeric behavior plays a pivotal role in conferring industrial application to this class of materials.

Abu-Omar and coworkers were the first to synthesize a diepoxy compound containing an imine group in its structure by condensation of vanillin with aminophenol and the following reaction of the diphenol product with epichlorohydrin [19]. As the concentration of dynamic groups in the network strongly affects the vitrimeric properties of the final material [20], increasing the number of imine groups embedded in the monomer structure represents an effective strategy to boost the exchange kinetic but also a way to increase the number of hydrolyzable sites. For example, Roig et al. synthesized a diimine-diglycidyl monomer from vanillin and 4,4′-oxydianiline and used different polyether amines as curing agents. A fast stress relaxation process, good mechanical recyclability, and a chemical degradability that strongly depends on the cross-linking density were obtained [21]. Weng and coworkers applied a similar concept for the development of a diimine–diphenol building block that was employed as a curing agent for epoxidized soybean oil [22]. Finally, Ding et al. obtained materials with flame retardant properties and high-impact strength associated with excellent mechanical and chemical recyclability, using 1,3-bis(3-aminopropyl) tetramethyl disiloxane in the synthesis of a diimine–diglycidyl monomer [23].

In the present work, a diepoxy building block containing two imine groups was synthesized with high yield from a commercial polyether diamine and the glycidyl derivative of vanillin. The rigid aromatic moieties introduced with vanillin and the flexible polyether spacer confer excellent mechanical properties and a simultaneous extremely fast thermal-induced stress relaxation process. Moreover, the absence of purification steps after the synthesis makes this synthetic process potentially scalable at the industrial level. Two different amines were studied as curing agents, resulting in different activation energies for the exchange process. The low viscosity of the diepoxy monomer at only 40 °C, together with the complete chemical degradability of the cured materials in an acid medium, make them promising for the development of new carbon fiber composite materials based on a vitrimeric matrix that potentially allows the total recovery of the carbon fibers.

## 2. Materials and Methods

### 2.1. Materials

The following chemicals were purchased from Sigma-Aldrich (Saint Louis, MO, USA): (±)-epichlorohydrin (ECH, ≥99%), poly(propylene glycol) bis(2-aminopropyl ether) (Jeffamine D-230, M_n_ ~ 230 g/mol), and 5-amino-1,3,3-trimethylcyclohexane-methylamine (isophorone diamine, IPDA, 99%, mixture of cis and trans). Benzyl triethylammonium chloride (TEBAC, 98%) was purchased from Alfa Aesar (Thermo Fisher, Kandel, Germany). *m*-Xylylene diamine (MXDA, ≥99%,) was purchased from TCI lmd (TCI Europe N.V. Zwijndrecht, Belgium). 4-Hydroxy-3-methoxy benzaldehyde (Vanillin, Van, 99%) was purchased from Thermo Fisher Scientific (Geel, Belgium). Sodium hydroxide (granulated, NaOH) and tetrahydrofuran (THF) were obtained from Scharlau (Barcelona, Spain). Dichloromethane (DCM) and ethyl acetate (EtOAc) were obtained from VWR Chemicals (West Chester, PA, USA). Anhydrous magnesium sulfate (MgSO_4_) was provided by Fluorochem (Hadfield, Glossop, UK). All chemicals were used as received.

### 2.2. Synthesis of Vanillin Glycidyl Ether (EVan)

EVan was prepared following a previously reported procedure [24,25]. A 250 mL three-neck round-bottom flask, equipped with a magnetic stirrer and reflux condenser, was charged with Van (7.6 g, 50.0 mmol), ECH (46.3 g, 500.0 mmol), and TEBAC (1.1 g, 5.0 mmol). The mixture was first stirred for 1.5 h at 80 °C and then cooled to room temperature. A mixed solution of TEBAC (1.1 g, 5.0 mmol) and NaOH (8.0 g, 200.0 mmol, 5.0 M) was added, and the mixture was stirred for 30 min at room temperature. After a complete conversion of Van, ethyl acetate and distilled water were poured into the mixture and stirred, and the aqueous phase was extracted three times with EtOAc. The organic phase was collected, dried over anhydrous MgSO_4_, and concentrated in a vacuum rotary evaporator. The residual amount of ECH was eliminated by dissolving the product in EtOAc and evaporating it under vacuum multiple times. The product was obtained as a yellowish-white solid with a 91% of yield (9.4 g). M.P.: 97.0 °C.

^1^H NMR (CDCl_3_, δ in ppm, Figure S1): 9.84 (s, 1H, H_1_), 7.42 (d, 1H, H_2_), 7.40 (s, 1H, H_4_), 7.02 (d, 1H, H_3_), 4.37 (dd, 1H, H_6_), 4.08 (dd, 1H, H_6_), 3.92 (s, 3H H_5_), 3.40 (m, 1H, H_7_), 2.91 (dd, 1H, H_8_), 2.77 (dd, 1H, H_8_).

^13^C NMR (CDCl_3_, δ in ppm, Figure S2): 190.9 (C_1_), 153.4 (C_8_), 150.0 (C_6_), 131.0 (C_2_), 126.5 (C_3_), 112.3 (C_4_), 109.5 (C_5_), 70.0 (C_9_), 56.0 (C_7_), 49.9 (C_10_), and 44.7 (C_11_).

### 2.3. Synthesis of the Diimine–Diglycidyl Ether Derivative (DIDE-PPO)

The diimine derivative of vanillin diglycidyl ether with Jeffamine D-230 (DIDE-PPO) was synthesized as follows: 1.68 g of Jeffamine D-230 was introduced into a 250 mL round-bottom flask. Then, 3.0 g of the previously prepared EVan was dissolved in 50 mL of DCM and added dropwise. The resulting mixture was kept under stirring at room temperature for 4 h. Subsequently, the solvent was removed under reduced pressure, and the product was dried at 60 °C under vacuum overnight. The desired product was obtained as a viscous oil that becomes solid at room temperature with quantitative conversion and was used without further purification.

^1^H NMR (CDCl_3_, δ in ppm, Figure S3): 8.18 (m, 2H, H_1_), 7.41 (d, 2H, H_2_), 7.13 (d, 2H, H_4_), 6.90 (d, 2H, H_3_), 4.26 (m, 2H, H_6_), 4.04 (m, 2H, H_6_), 3.91 (s, 6H, H_5_), 3.86–3.38 (m, H_7_ + PPO chain), 2.89 (m, 2H, H_8_), 2.74 (m, 2H, H_8_), 1.21–0.97 (m, -CH_3_ PPO chain).

^13^C NMR (CDCl_3_, δ in ppm, Figure S4): 160.0 (C_1_), 150.1 (C_8_), 149.7 (C_6_), 130.6 (C_2_), 122.7 (C_3_), 113.0 (C_4_), 109.8 (C_5_), 76.1–73.8 (PPO chain), 70.1 (C_9_), 65.7 (C_12_), 56.1 (C_7_), 50.1 (C_10_), 45.0 (C_11_), 19.0 (C_13_), 17.4 (C_14_).

### 2.4. General Procedure for the Preparation of Vitrimeric Samples

The synthesized DIDE-PPO was mixed with *m*-xylylene diamine (MXDA) or isophorone diamine (IPDA). In a typical procedure, 3.0 g of DIDE-PPO was mixed with a stoichiometric proportion of diamine (epoxide/amine group ratio 2:1 mol/mol). Because of the high viscosity of DIDE-PPO, it was first dissolved in 10 mL of DCM in a round-bottom flask and mixed with the diamine. The solvent was then evaporated under reduced pressure. The formulation was poured into a rectangular Teflon mold of 30.0 × 5.0 × 1.5 mm^3^ and heated at 35 °C for 30 min in a vacuum oven to remove residual solvent and trapped air. The samples were finally cured in an oven using the following schedule: 3 h at 100 °C, 3 h at 150 °C, and finally 2 h at 180 °C.

### 2.5. General Procedure for the Preparation of Composite Materials

The composite materials were prepared using carbon fiber five harness satin (5HS) woven fabric with a surface weight of 280 g/m^2^, supplied by Hexcel. In a typical procedure, 6.0 g of the formulation, stored at 40 °C to keep it liquid, was poured in a rectangular Teflon mold of 6.0 × 4.0 cm, and the carbon fiber was impregnated with the help of a spatula. Another 2 sheets of carbon fibers were added, repeating the same impregnation procedure. The sample was then kept at 50 °C for 30 min to reduce the resin viscosity and allow a better permeation through the carbon fibers. Finally, the curing was completed in a hot-press at 100 °C for 3 h, followed by 3 h at 150 °C and 2 h more at 180 °C, maintaining a pressure of 6 MPa. Once completely cured, the sample was left to cool down and cut off to obtain a square sample.

### 2.6. Characterization Techniques

^1^H NMR and ^13^C NMR spectra were recorded in a Varian VNMR-S400 NMR (Varian, Lake Forest, CA, USA) spectrometer. CDCl_3_ was used as the solvent. All chemical shifts are quoted on the δ scale in part per million (ppm) using residual protonated solvent signal as the internal standard (^1^H NMR: CDCl_3_ = 7.26 ppm; ^13^C NMR: CDCl_3_ = 77.16 ppm).

The thermal stability of the materials was evaluated using a Mettler Toledo TGA 2 (Mettler, Columbus, OH, USA) thermobalance. Cured samples weighing around 10 mg were degraded between 30 and 600 °C at a heating rate of 10 °C·min^−1^ in N_2_ atmosphere with a flow rate of 50 cm^3^·min^−1^.

The thermomechanical properties were studied using a DMTA Q850 (TA Instruments, New Castle, DE, USA) equipped with a film tension clamp. Prismatic rectangular samples with dimensions of around 30.0 × 5.0 × 1.5 mm^3^ were analyzed from −10 to 180 °C at 1 Hz, 0.1% strain, and a heating rate of 2 °C·min^−1^. Tensile stress relaxation tests were conducted in the same instrument using the film tension clamp on samples with the same dimensions as previously defined. The samples were first equilibrated at the relaxation temperature for 5 min, and a constant strain of 1% was applied, measuring the consequent stress level as a function of time. The materials were tested only once at each temperature.

Frequency sweep tests were performed in the same DMTA Q850 instrument equipped with the tensile geometry applying an oscillation strain of 0.05%. The frequency was swept from 10 to 0.05 Hz, collecting 4 data points per decade for each temperature. Temperature ranged between 130 and 170 °C.

To determine the viscosity at each temperature, a series of creep experiments were carried out with the same DMA Q850 instrument equipped with the film tension clamp at temperatures between 40 and 180 °C, increasing 10 °C in each scan. To perform the tests, the samples were equilibrated for 5 min at the selected temperatures, and a stress level of 0.1 MPa was applied for 30 min. The viscosity η (Pa·s) was obtained from the creep plots: first, the strain rate (ἐ) was determined from the slope of the ε-time curves, and then the viscosity was calculated using the following expression:

(1) $$\eta =\frac{\sigma }{\dot{\varepsilon }}$$

Creep tests at service temperature were also conducted in the DMA with a tensile geometry. The sample was equilibrated at 30 °C for 5 min, after which the stress (*σ*) was applied for 30 min and then released. The strain (ε) was measured as a function of time during the application of the stress and for an additional 30 min for the stress recovery. The applied stress was chosen, ensuring that each material was in its viscoelastic range and producing a deformation of 0.1%.

The chemical degradation of the vitrimeric materials was conducted by first weighing the samples of the cured material and then soaking them in a 2 M HCl and THF solution (2/8 *v*/*v*) for 5 h at 60 °C under stirring. Regarding the carbon-fiber-reinforced composite materials, the chemical degradation of the polymer matrix was conducted in the same condition previously mentioned. The carbon fibers were then recovered by filtration and washed with THF, water, and finally acetone. The residual carbon fibers were then dried at 90 °C.

Environmental scanning electron microscopy (ESEM) by means of a FEI ESEM Quanta 600 instrument (Hillsboro, OR, USA) was used to analyze the morphology of carbon fibers before and after carrying out the chemical degradation of the composites. Electrons were accelerated at 20.00 kV.

## 3. Results and Discussion

### 3.1. Synthesis of the Diimine Diepoxy Monomer DIDE-PPO

**Scheme 1:**
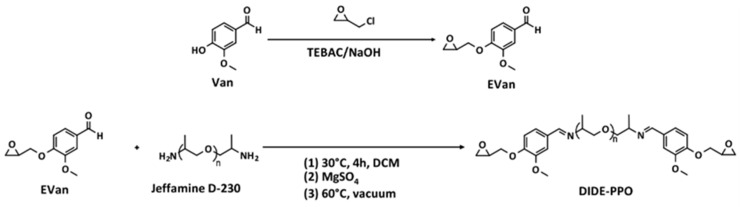
Synthetic procedure for the synthesis of the diglycidyl imine monomer (DIDE-PPO).

The diimine diepoxy monomer (DIDE-PPO) was synthesized following an already reported procedure consisting of two stages [26]. In the first one, the glycidyl derivative of vanillin (EVan) was synthesized, followed by the condensation reaction with Jeffamine D-230 (Scheme 1).

EVan was synthesized by reacting vanillin with a large excess of epichlorohydrin, using NaOH as a base to promote the oxirane ring closure and TEBAC as a phase transfer catalyst maintaining the mixture for 1.5 h at 80 °C. The procedure led to the formation of the expected glycidyl product as a yellowish-white solid with high yield. Its structure was confirmed by ^1^H NMR spectroscopy by the appearance of the signals corresponding to the glycidyl group between 4.37 and 2.77 ppm, while the formyl proton signal remained unaltered (Figure S1). ^13^C NMR spectrum showed the expected signals of the carbons corresponding to the glycidyl group between 70.0 and 44.7 ppm (Figure S2).

After that, the condensation reaction of Jeffamine D-230 with a stoichiometric amount of EVan was conducted for 4 h at 30 °C to prevent the competitive reaction between epoxides and amine groups. Then, the water produced in the condensation reaction was removed by adding anhydrous MgSO_4_, and the equilibrium was displaced to form the imines. In this way, after solvent elimination by rotary evaporation under vacuum at 60 °C, a quantitative conversion was reached, and the product was obtained as a viscous, yellowish oil that rapidly solidified at room temperature. Finally, it was characterized by ^1^H and ^13^C NMR spectroscopy (Figures S3 and S4). The appearance of the signal at 8.18 ppm revealed the formation of the imine group. The comparison between its integral and the integral of the signal of the aldehyde group of EVan confirmed almost complete conversion (98%).

### 3.2. Study of the Curing Procedure

The curing procedure employed for the prepared formulations consisted of a thermal treatment at 100 °C for 3 h, followed by 3 h at 150 °C and a post-curing of 2 h at 180 °C to ensure the full reaction of the oxirane rings with the amine groups.

**Figure 1:**
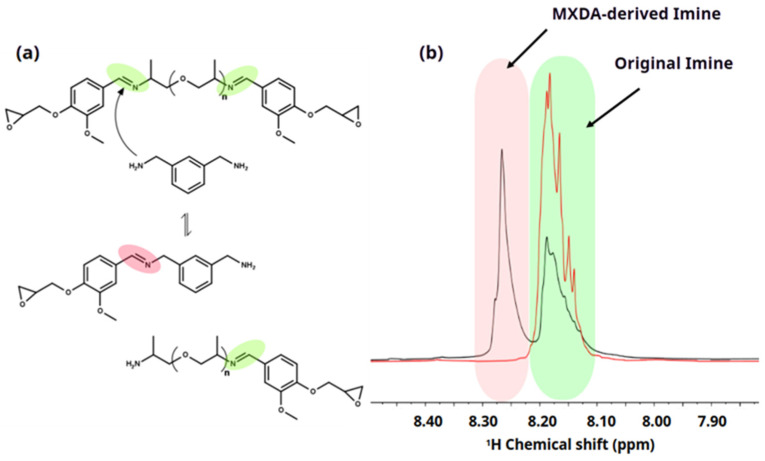
(**a**) Scheme of the side transamination reaction during the curing process of DIDE-PPO with MXDA. The green color corresponds to the initial imine and pink color to the new imine group formed by transamination reaction. (**b**) Signals of the imine group protons from the ^1^H NMR spectra of the reaction mixture. The green color region corresponds to the signal attributed to the initial imine before and after the transamination reaction while the pink color region corresponds to the signal attributed to the new imine group formed by transamination reaction.

Considering the chemical structure of the synthesized diepoxy monomer DIDE-PPO and the amine hardeners, side reactions during the curing process should be considered. In this specific case, transamination reactions between primary amines from the hardener and imine groups of DIDE-PPO could determine the formation of new imine groups and the release of new primary amines. Figure 1a shows the scheme of the possible reaction between an imine group contained in the diepoxy monomer and the primary amines of MXDA hardener.

^1^H NMR spectroscopy characterization conducted on the formulation heated for 10 min at 40 °C, to avoid gelation, confirms that transamination reactions had taken place. The appearance of a signal at 8.27 ppm (Figure 1b) reveals the formation of new imine groups in addition to the original imine signal at 8.18 ppm of the DIDE-PPO monomer. Longer reaction times did not affect the area of the signals of the two imine groups, revealing that the equilibrium was already reached in 10 min. The effect of the temperature on the evolution of the transamination reaction was monitored, increasing the temperature to 60 °C and then to 100 °C, and the ^1^H NMR spectra was collected after 10 min at each temperature. Figure S5 shows the superimposition of the whole spectra of the formulation at different reaction temperatures (40, 60, and 100 °C). The signal at 4.77 ppm related to the protons of the methylene group of MXDA directly linked to an imine group corroborates the thesis that a transamination reaction between epoxy monomer and hardener occurs since there is a slight displacement of its chemical shift and the signal becomes broader due to the loss of symmetry.

The amine curing agent was added in a stoichiometric ratio (1:1) between glycidyl and -NH- groups, meaning a molar proportion of 2:1 DIDE-PPO/diamine curing agent because of the functionality equal to 4 of the diamines employed in the epoxy–amine reaction. Considering that every diglycidyl molecule contains two imine groups in its structure, a quantitative conversion of the primary amines of the curing agent in the transamination reaction would produce a drop of the original imine content of the 50% and the formation of an equal amount of new imine groups produced by the hardener. Therefore, in the case of quantitative conversion, a ratio of 1 between the integrals of the two imine signals is expected in the ^1^H NMR spectrum of the mixture.

Observing the spectra in Figure S5, the ratio between the integrals of the two imine signals, collected in Table S1, slightly increased with the temperature, from 0.82 at 40 °C to 0.89 at 100 °C. It reveals an important conversion of the hardener amine groups into imine moieties in the epoxy monomer, that at 100 °C was close to 90%. Higher temperatures or longer reaction times could not be investigated because of the occurrence of gelation and the consequent insolubility of the product.

According to these results, the polymer network embeds two different aliphatic imine groups: the original one, contained in the diglycidyl monomer and derived by the reaction of vanillin and Jeffamine D-230, and the imine derived by the transamination reaction of the existing imine groups and the amine groups of the hardener. As examined in depth in Section 3.5, this double dynamic system affects the stress relaxation behavior of the materials, even if the two different relaxation paths occur through imine metathesis reaction.

### 3.3. Thermal Stability Characterization of the Materials

The thermal stability of the cured samples was studied by thermogravimetric analysis (TGA). Figure S6a shows the TGA curves of each formulation, while Figure S6b shows the first derivative of the TGA curves. All the thermal degradation data are summarized in Table 1. The temperature of 1% and 2% weight loss and the temperature of the maximum degradation rate were very similar for both materials. These values allow a safe recycling or remolding of the materials up to 273 °C. The main difference was observed in the char yield at 600 °C where the aromatic ring of the curing agent MXDA played a key role, increasing the char yield from 27.7% in the case of DIDE-PPO/IPDA to 37.9% in DIDE-PPO/MXDA.

### 3.4. Thermo-Mechanical Properties of the Materials

**Figure 2:**
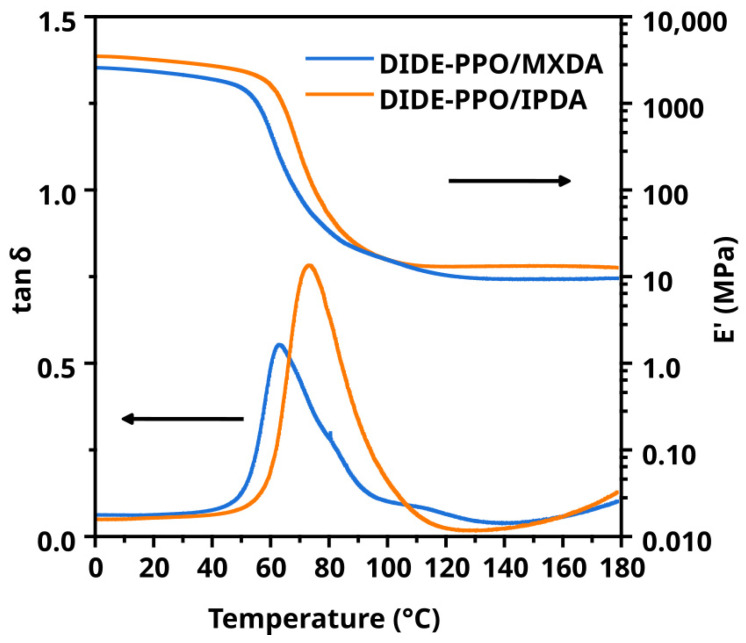
Evolution of storage modulus and tan δ with temperature for both materials.

Thermomechanical properties were determined by DMA. Figure 2 shows the storage modulus (E′) and tan δ as a function of temperature for the prepared materials. The main thermomechanical data obtained from those experiments are collected in Table 1.

DIDE-PPO/IPDA and DIDE-PPO/MXDA exhibited T_tan δ_ of 73 and 63 °C, respectively. The material cured with MXDA showed a lower T_tan δ_ despite the curing agent bearing an aromatic ring capable of noncovalent π–π interaction. It is reasonable that the methylene carbon between the amine groups and the aromatic ring conferred high mobility to the network at a molecular scale. From the other side, IPDA did not bear any aromatic ring, but one of the amine groups was directly linked to the cycloaliphatic moiety. It determines a more hindered network and consequently higher T_tan δ_ values. The broadness of the peaks is ascribable to the network inhomogeneity produced by the transamination reaction before and during the curing process, as described in Section 3.2. Finally, the progressive growth of the tan δ values starting from 130 °C was due to the stress relaxation process occurring during the measurement. Indeed, as will be discussed in Section 3.5, stress relaxation was extremely fast in this range of temperatures for both synthesized materials, and the relaxation times were comparable to the oscillation period of the isofrequency temperature ramp experiment. It means that relaxation processes occurred during the oscillation period, and they were detected by the instrument. This was confirmed by isothermal frequency sweep experiments conducted between 130 and 170 °C, where a drop of the storage modulus occurred, increasing the temperature at 1 Hz (6.28 rad/s), as shown in Figure S7 for both materials. At higher frequencies, the temperature increase produced a slighter drop of E′, indicating that the oscillation period was in the same order of magnitude of the stress relaxation times.

Regarding the storage modulus in the rubbery state, E′_Rubbery_, a slightly higher value was observed for DIDE-PPO/IPDA, despite the two curing agents presenting the same functionality. It is ascribable to the IPDA structure, which was more compact and led to a smaller distance between crosslinks. It also determined a more rigid structure than MXDA, where the methylene group located between the amine groups and the aromatic ring increased the network mobility, resulting in a lower storage modulus in the glassy state, E′_g_.

### 3.5. Vitrimeric Properties

**Figure 3:**
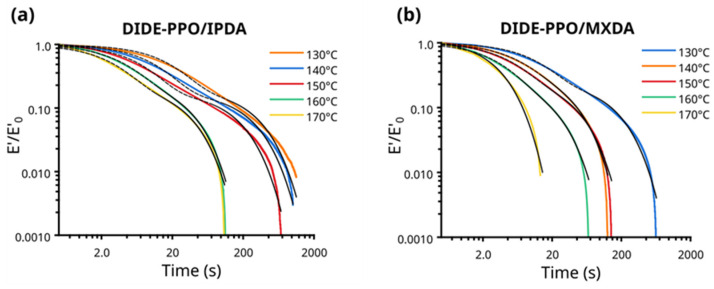
Tension mode stress relaxation profiles (colored lines) and the corresponding fitting by a double-mode Maxwell function (dashed black lines) of (**a**) DIDE-PPO/IPDA and (**b**) DIDE-PPO/MXDA.

The vitrimeric behavior of the synthesized materials was first investigated by stress relaxation experiments conducted in tension in a range of temperatures between 130 and 170 °C. The evolution of the normalized stress was monitored as a function of time, and the stress relaxation profiles obtained are shown in Figure 3. DIDE-PPO/MXDA and DIDE-PPO/IPDA exhibited very fast stress relaxation processes, reaching total relaxation in less than 12 min and 18 min, respectively, at 130 °C, the lower temperature tested. At 170 °C, the material was totally relaxed at 14 s in the case of DIDE-PPO/MXDA and 100 s for DIDE-PPO/IPDA, potentially demonstrating fast moldability and reprocessability.

The normalized stress relaxation profiles were firstly fitted to the Kohlrausch–Williams–Watts (KKW) stretched exponential decay function:

(2) $$\frac{E^{\prime }}{{E_{0}}^{\prime }}=\exp{\left(\frac{-t}{\tau }\right)}^{\beta }$$

The KWW model is known to usually describe quantitatively the stress relaxation process of vitrimers and polymers in general [27,28,29,30], considering the distribution of overlapping relaxation modes that contribute to the macroscopic stress relaxation of the material [31]. Specifically, the breadth of the relaxation distribution is described in the model by the stretching parameter β (0 ≤ β ≤ 1), while τ is the characteristic relaxation time and t is the decay time.

Figure S8 shows the fits by KWW functions superimposed to the stress relaxation profiles for both materials, while Table S2 groups the fitting parameters and the coefficients of determination (R^2^). The stretched parameter β ranged from 0.41 to 0.62 and from 0.49 to 1.00 for DIDE-PPO/IPDA and DIDE-PPO/MXDA, respectively, reflecting broad relaxation distribution for both materials. It is evident from the R^2^ values and qualitatively from the observation of the shapes of the fits with respect to the experimental data that the stretched exponential decay function does not precisely describe the relaxation process of the systems object of this study.

The involvement of the curing amine in transamination reactions before the gel point and the consequent formation of two different types of imine groups, as described in Section 3.2, suggested the possibility of multimodal stress relaxations. Although the formed imine groups should exhibit similar reactivity, as both are derived from the reaction of an aliphatic amine with an aromatic aldehyde, the existence of two distinct imine groups characterized by different mobility in the network could affect the distribution of relaxation modes.

A multi-mode Maxwell model constituted of two Maxwell elements arranged in parallel was then considered to describe the behavior of these systems during the stress relaxation processes. In this case, the normalized relaxation modulus is described by the linear combination of two decaying exponential functions:

(3) $$\frac{E^{\prime }}{{E_{0}}^{\prime }}=\mathrm{Aexp}\left(\frac{-t}{\tau_{1}}\right)+\left(1-A\right)\exp\left(\frac{-t}{\tau_{2}}\right)$$

The equation determines two characteristic relaxation times τ_1_ and τ_2_ related to the two Maxwell elements of the model, while A is the pre-exponential factor. Figure 3 shows the fits of Equation (3) superimposed on the experimental stress relaxation profiles, while the fitting parameters and the coefficients of determination are provided in Table 2.

As evidenced by the R^2^ values obtained from the fitting, the linear combination of two exponential functions was found to adjust the experimental stress relaxation data finely.

**Figure 4:**
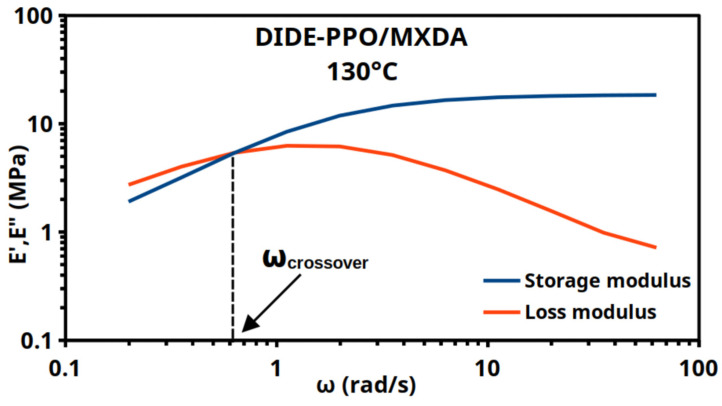
Frequency sweeps for DIDE-PPO/MXDA at 130 °C show the crossover of E′ and E″.

Frequency sweep experiments were also conducted at the same range of temperatures to corroborate the results obtained through stress relaxation experiments. Because an oscillatory test obtains multiple measurements of the same material features at each step, providing more reliable data and a better signal-to-noise ratio [32], the frequency sweep experiments represented a trustworthy choice. Frequency sweeps tests were performed, and the characteristic relaxation time was determined from the intersection point of the storage (E′) and loss moduli (E″) (Figure 4). Specifically, for frequency data collected in angular form (units of rad/s), the crossover time was determined as τ_crossover_ = 2π/ω_crossover_.

Figures S9 and S10 show the frequency sweep experiments for the studied materials for all the temperatures tested.

Comparing the characteristic timescales obtained from frequency sweep (Table 2) and the stress relaxation data, it is possible to observe an agreement in the trends of τ_crossover_ and *τ_2_*. Frequency sweep crossover times (τ_crossover_) are generally higher than the characteristic times obtained from stress relaxation experiments, as previously reported by Porath et al. [32]. This is true in particular for the sample DIDE-PPO/IPDA, while DIDE-PPO/MXDA exhibited crossover times in agreement with the stress relaxation experiments. Moreover, in frequency sweep experiments, it is not possible to discern the two different relaxation modes, and τ_crossover_ can be considered the overall result of both of them, where the slow relaxation mode affects the total relaxation time resulting in slightly longer times compared to the fast relaxation element τ_2_.

**Figure 5:**
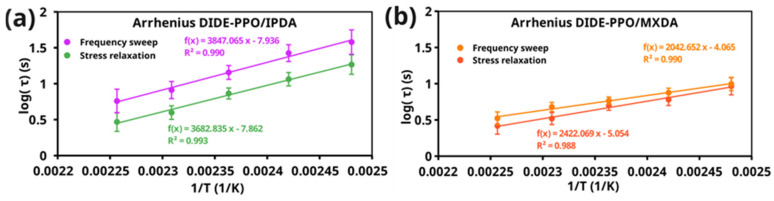
Arrhenius plots obtained from the crossover times (from frequency sweep experiments) and the characteristic relaxation times of the short-time stress relaxation mode from the stress relaxation experiments of (**a**) DIDE-PPO/IPDA and (**b**) DIDE-PPO/MXDA.

The Arrhenius relationship was deduced from the characteristic relaxation times obtained with both methods by fitting log(τ_crossover_) and log(τ_2_) as a function of the inverse of the temperature, and the apparent activation energies E_a_ for the stress relaxation process were calculated for each formulation. Figure 5 shows the Arrhenius plots of the studied materials obtained by stress relaxation and frequency sweep experiments.

The relaxation times obtained from the two methods exhibited very similar temperature dependencies, those reflected in concordant values of the apparent activation energies for the imine metathesis exchange reaction (Table 3). The dynamic exchange in DIDE-PPO/IPDA materials was characterized by a higher activation energy compared to DIDE-PPO/MXDA. It could be ascribed to network effects. Indeed, as discussed in Section 3.4, the IPDA curing agent conferred less mobility to the network than MXDA. It could result in a lower mobility of the imine groups and, consequently, in a higher energy barrier for the exchange reaction. Moreover, one of the amine groups in the isophorone structure was directly attached to the cyclohexane ring, creating steric hindrance due to substituents at the 3 and 5 positions. This made it difficult for another imine group to approach and exchange, resulting in higher relaxation times and higher activation energy.

### 3.6. Chemical Degradation

As previously mentioned, imine groups can be hydrolyzed in acid conditions. Therefore, the presence of this chemical group in a three-dimensional polymer network opens the possibility to depolymerize the matrix to smaller soluble units. The accessibility of the degradable groups with respect to the acid medium is crucial for the outcome of the degradation process. In this context, a fundamental role is played by the diffusion of water and acid catalyst in the material structure. An organic solvent is usually added to the degradation system to favor the swelling of the polymer matrix and the penetration of water [33]. Previous studies revealed that THF is a suitable solvent to facilitate the penetration of the H_3_O^+^ into a polyimine crosslinked structure, increasing the wettability of the polymer surface [34].

**Figure 6:**
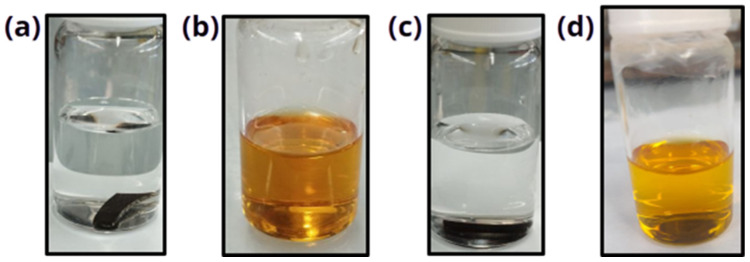
Chemical degradation of the prepared materials: DIDE-PPO/MXDA before (**a**) and after (**b**) chemical degradation, and (**b**) DIDE-PPO/IPDA before (**c**) and after (**d**) chemical degradation.

In this study, the chemical degradability of the prepared materials was investigated at 60 °C for 5 h using a mixture of 2 M HCl and THF (2/8 *v*/*v*). The results obtained during the chemical degradation tests for all the vitrimeric materials are shown in Figure 6. Both materials totally degraded in 5 h, demonstrating that their use in the preparation of carbon-fiber-reinforced composites (CFRC) would allow the recovery of the fibers by degradation of the polymer matrix in a recycling process after their end of life.

### 3.7. Creep Experiments

**Figure 7:**
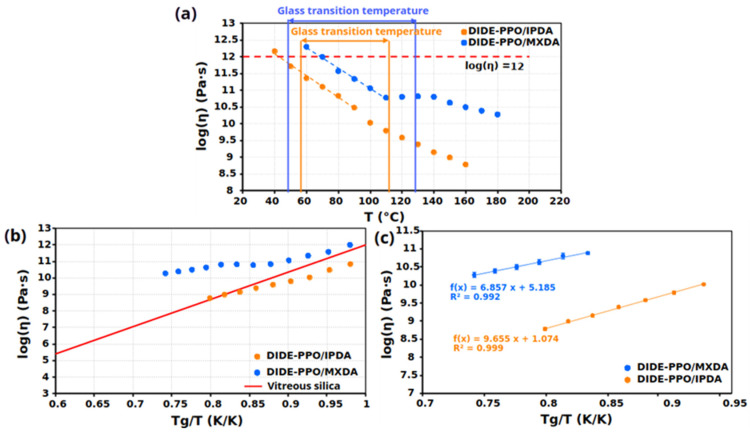
(**a**) Viscosity as a function of the temperature for the prepared materials. (**b**) Angell fragility plot for all the prepared materials. (**c**) Determination of Arrhenius apparent activation energy of creep viscosity for the prepared materials.

Creep tests were conducted on the synthesized materials at temperatures well below their T_g_. The logarithm of the viscosity (the inverse of the slope of the ε-t curves) was then plotted against the temperature for all samples (Figure 7a).

Both materials exhibited two different regimes ascribable to different phenomena responsible for the viscous behavior of the materials. While during the transition of the T_g_ the segmental motion, the processes described by the William–Landel–Ferry (WLF) model are mainly responsible for the network rearrangement, at higher temperatures, the exchange reactions between dynamic groups control the network rearrangement [35,36]. This results in two different temperature dependencies: one within the glass transition temperature range and another above it.

The topology freezing temperature (T_v_) can be calculated from these plots since, by convention [37], it is the temperature at which the material reaches a viscosity of 10^12^ Pa⋅s. However, it is also considered the temperature at which chemical interchanges start to occur. The T_v_s were calculated by extrapolation from the graphs and are collected in Table 4, and for both materials result lower than the T_g_. According to this, exchange reactions can only occur when the T_g_ is overpassed and the network acquires sufficient mobility to permit dynamic group interactions. For this reason, for the studied materials, the T_g_ represents the practical lower limit beyond which the imine metathesis reactions occur.

Angell fragility plots were represented by plotting the viscosity as a function of the inverse of the temperature normalized to the T_g_ (Figure 7b). As expected, both materials presented a gradual decrease in viscosity when increasing the temperature. In particular, viscosity exhibited a linear dependency with respect to T_g_/T following an Arrhenius law at temperatures above the glass transition temperature. Considering this, above T_g_, the viscous behavior of these materials was dominated by the exchange process, and the viscous flow activation energy can be calculated from the linear regression of this plot (Figure 7c). The activation energies obtained from creep tests, collected in Table 4, strongly agrees with the activation energies that result from stress relaxation and frequency sweep experiments, providing relevant consistency to the obtained results. Based on the calculated activation energies, network mobility played a determinant role in the creep behavior of the two materials. Similarly to what was observed in stress relaxation results, DIDE-PPO/IPDA exhibited higher activation energy because of the higher network rigidity conferred by the IPDA hardener and the steric effects. On the other hand, MXDA determined the formation of a less hindered network due to the higher mobility introduced by the methylene carbon between the nitrogen atom and the aromatic ring.

### 3.8. Carbon-Fiber-Reinforced Composite Materials

The use of DIDE-PPO/MXDA and DIDE-PPO/IPDA in the preparation of carbon-fiber-reinforced composite materials was investigated.

**Figure 8:**
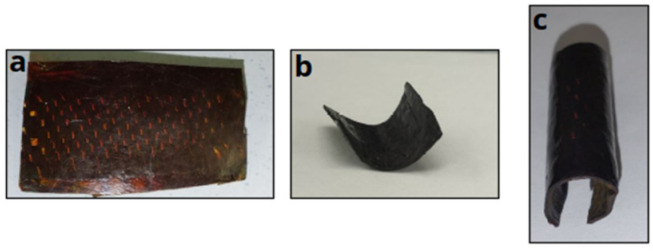
(**a**) Carbon-fiber-reinforced composite made using DIDE-PPO/IPDA before reshaping in its completely cured state, (**b**) after reshaping at 170 °C in a cylindrical mold of 28 mm of diameter, and (**c**) after reshaping at the same temperature in a cylindrical mold of 16 mm diameter.

The composites were prepared by impregnation of three layers of carbon fiber twill, as described in Section 2.5. According to the procedure, the materials were completely cured, differently from the typical procedure followed in the preparation of preimpregnated materials (prepreg). Indeed, traditional prepreg are only partially cured, then reshaped to their final form and finally completely cured [38,39]. This procedure introduces issues and costs regarding the storage of the material before the reshaping to prevent its premature curing [40]. From this point of view, vitrimer-based composites overcome the problem, exhibiting reshapeability, even if completely cured thanks to the presence of dynamic bonds that allow stress relaxation processes at specific temperatures. Figure 8 shows the composite prepared using DIDE-PPO/IPDA before (Figure 8a) and after (Figure 8b,c) reshaping in its completely cured state.

The reshaping was conducted after a conditioning step at 170 °C for 15 min, bending the composite sheet and introducing it in cylindrical molds of different diameters at this temperature, relaxing the internal stress and thus generating a permanent deformation. This result demonstrates that composite materials prepared from vitrimeric systems based on the monomer DIDE-PPO can be easily processed after total curing.

As expected, the introduction of carbon fibers in the prepared vitrimers determines an improvement of the mechanical properties. Specifically, Figure S11 shows the evolution of storage modulus E′ and tan δ with temperature conducting the tests in a three-point bending geometry. As reported in Table S3, a great increase in the storage modulus and a broadening of tan δ evolution was observed for both materials, although the fiber did not affect the position of the maximum of the tan δ peak.

**Figure 9:**
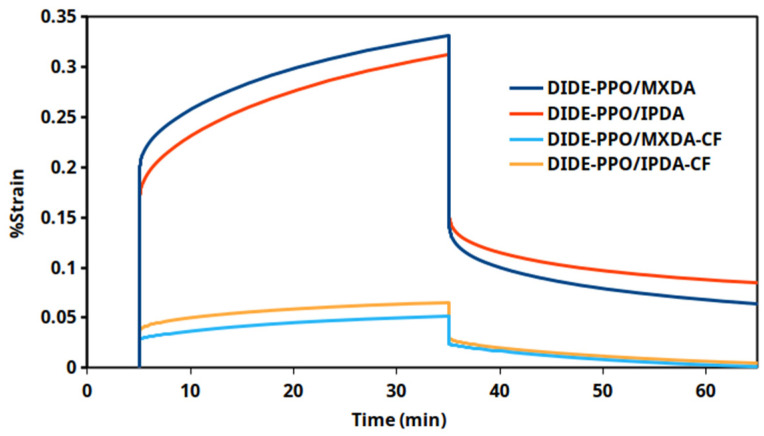
Creep test conducted at 30 °C for the prepared vitrimeric materials and the related CFRC.

Creep tests at 30 °C were also conducted on the vitrimers DIDE-PPO/MXDA and DIDE-PPO/IPDA and on the related carbon-fiber-reinforced materials. In Figure 9, the strain profiles and the following strain recovery as a function of time for the different materials are compared, while in Table S4, the creep rate and the strain recovery values are collected.

The use of carbon fibers within the vitrimeric matrix clearly improved the creep resistance of the materials, determining a reduction of the creep rates of an order of magnitude. Moreover, both composites exhibited faster and complete strain recovery when the stress was released compared to the non-reinforced vitrimeric systems, which exhibited poor strain recovery capability. These results suggest a considerable effect of the carbon fibers in preventing plasticization phenomena. Therefore, the creep susceptibility of the pristine vitrimeric materials based on the monomer DIDE-PPO does not preclude the use of these systems in the preparation of composite materials for applications where creep resistance is demanded.

**Figure 10:**
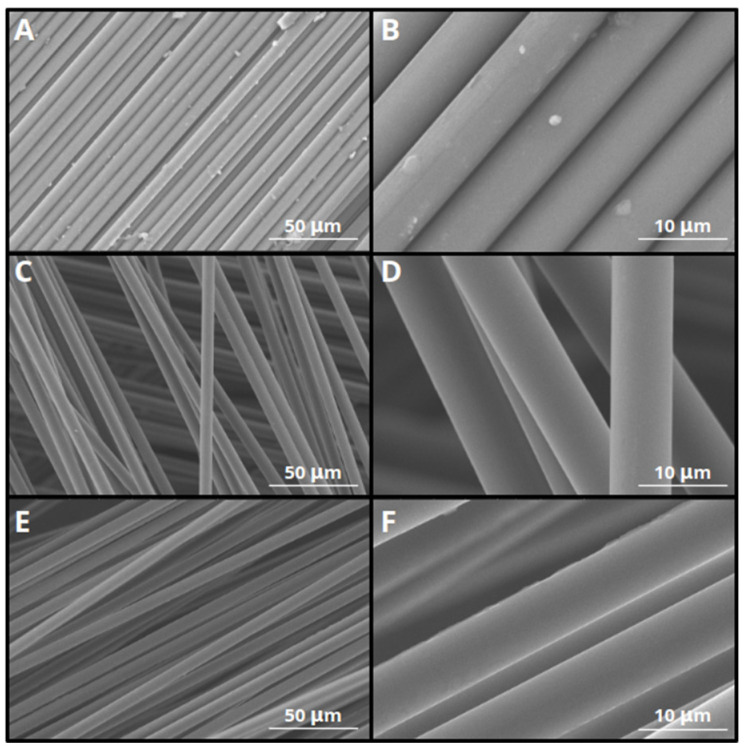
ESEM images of pristine carbon fibers at (**A**) magnification ×1000 and (**B**) magnification ×5000; carbon fibers after acid degradation of DIDE-PPO/IPDA at (**C**) magnification ×1000 and (**D**) magnification ×5000; and carbon fibers after acid degradation of DIDE-PPO/MXDA at (**E**) magnification ×1000 and (**F**) magnification ×5000.

We finally verified that by hydrolyzing the vitrimeric matrix in acidic medium, setting up the conditions described in Section 2.6., the carbon fibers could be recovered. Figure 10 shows the ESEM micrographs of the original carbon fiber twill and the same twill recovered after the chemical degradation of the matrix.

As evidenced by the micrographs, chemical degradation allowed for the complete removal of the polymeric matrix and did not produce any physical damage to the carbon fibers, making their recovery and reuse possible.

## 4. Conclusions

A diepoxy monomer containing two imine groups was able to be obtained from the glycidyl derivative of vanillin and Jeffamine D-230, a commercial polyether diamine, through an easy and scalable synthetic procedure. By curing it with different amine hardeners, vitrimeric systems exhibiting a very fast stress relaxation process and high temperature stability were obtained.

Side transamination reactions between the amine curing agents and the imine groups of the diepoxy monomer were observed before gelation, and the formation of new imine groups was confirmed by means of ^1^H NMR spectroscopy.

The presence of two different imine groups in the network was found to affect the stress relaxation profiles. The analysis of the experimental data using a double-element Maxwell model confirmed that the relaxation process was the result of the combination of two relaxation modes characterized by different relaxation times.

From both stress relaxation and creep experiments, a strong dependency of the activation energy of the exchange reaction with respect to the used hardener was observed. In particular, IPDA determines the formation of a more hindered network where the dynamic group mobility is reduced, and imine metathesis is consequently characterized by higher activation energy. Therefore, the choice of the hardener not only impacts the thermo-mechanical properties but also allows the tuning of the stress relaxation kinetics.

Both studied materials exhibited total chemical degradability in acidic conditions, opening up the possible use of these vitrimers in the preparation of composite materials where a recovery of the reinforcement is required. Specifically, we prepared carbon-fiber-reinforced materials using both vitrimers (DIDE-PPO/IPDA and DIDE-PPO/MXDA) and proved that the carbon fibers could be recovered by acid treatment without observing any structural damage to the fibers.

Triggering the imine metathesis reaction with temperature, easy reshape could be performed, even after the complete curing of the polymer matrix thanks to the extremely fast stress relaxation of the studied vitrimers.

The prepared composites finally showed a great creep resistance, demonstrating their possible use in applications where low deformation is a requirement.

## Figures and Tables

**Table 1:** Thermal and thermo-mechanical data of the prepared materials.

Sample	T_1%_ ^a^ (°C)	T_2%_ ^b^ (°C)	T_max_ ^c^ (°C)	Char Yield ^d^ (%)	T_tan δ_ ^e^ (°C)	FWHM ^f^ (°C)	E′_Glassy_ ^g^ (MPa)	E′_Rubbery_ ^h^ (MPa)
DIDE-PPO/IPDA	275 ± 1	289 ± 2	342 ± 5	27.7 ± 0.5	73.0 ± 3.0	24 ± 4	3117 ± 80	13 ± 2
DIDE-PPO/MXDA	273 ± 3	286 ± 3	343 ± 4	37.9 ± 1.0	63.0 ± 2.0	25 ± 3	2437 ± 95	9 ± 1

^a^ Temperature of 1% of weight loss. ^b^ Temperature of 2% of weight loss. ^c^ Temperature of the maximum degradation rate. ^d^ Char residue at 600 °C. ^e^ Temperature at the maximum of tan δ peak at 1 Hz. ^f^ Full width at half maximum of tan δ peak at 1 Hz. ^g^ Storage modulus in the glassy state (at T_tan δ_ − 50 °C). ^h^ Storage modulus in the rubbery state (at T_tan δ_ + 50 °C).

**Table 2:** Fitted parameters for the double-mode Maxwell equation and crossover times for the prepared materials.

Sample	T (°C)	A ^a^	τ_1_ ^b^ (s)	τ_2_ ^c^ (s)	R^2^	τ_crossover_ ^d^ (s)
DIDE-PPO/IPDA	130	0.797	308.74	18.45	0.992	37.92
	140	0.18	258.01	11.54	0.991	26.6
	150	0.181	160.08	7.31	0.993	14.33
	160	0.271	27.93	3.96	0.996	8.16
	170	0.227	26.87	2.94	0.991	5.74
DIDE-PPO/MXDA	130	0.302	155.54	9.21	0.992	9.91
	140	0.619	34.03	6.03	0.999	7.51
	150	0.719	39.77	5.01	0.998	5.82
	160	0.73	18.99	3.3	0.993	4.77
	170	0.231	3.79	2.62	0.989	3.33

^a^ Pre-exponential factor. ^b^ Characteristic relaxation time related to the slower relaxation mode. ^c^ Characteristic relaxation time related to the faster relaxation mode. ^d^ Crossover time from frequency sweep experiments.

**Table 3:** Adjusting parameters from Arrhenius plots built from stress relaxation and frequency sweep experiments.

	Stress Relaxation			Frequency Sweep		
Sample	E_a_ (kJ/mol)	logA (s)	R^2^	E_a_ (kJ/mol)	logA (s)	R^2^
DIDE-PPO/IPDA	70 ± 11	7.9 ± 1.3	0.993	74 ± 13	7.9 ± 1.6	0.99
DIDE-PPO/MXDA	46 ± 9	5.0 ± 1.2	0.988	39 ± 7	4.1 ± 0.9	0.99

**Table 4:** Vitrimeric properties and adjusting parameters obtained from the Arrhenius plot of the creep viscosity for the prepared materials.

Sample	T_v_ ^a^ (°C)	E_a_ (kJ/mol)	logA (s)	R^2^
DIDE-PPO/IPDA	43	64 ± 2	1.1 ± 0.3	0.999
DIDE-PPO/MXDA	71	44 ± 5	5.2 ± 0.6	0.992

^a^ Topology freezing temperature obtained from the Arrhenius relationship.

## Data Availability

The original contributions presented in the study are included in the article/Supplementary Materials, further inquiries can be directed to the corresponding author.

## Supplementary Materials

The following supporting information can be downloaded at: https://www.mdpi.com/article/10.3390/polym16192754/s1, Figure S1. ^1^H NMR spectrum of EVan in CDCl_3_; Figure S2. ^13^C NMR spectrum of EVan in CDCl_3_; Figure S3. ^1^H NMR spectrum of DIDE-PPO in CDCl_3_; Figure S4. ^13^C NMR spectrum of DIDE-PPO in CDCl_3_; Figure S5. ^1^H NMR spectra in CDCl_3_ of the curing mixture at 40, 60 and 100 °C collected after 10 min of equilibration at each temperature; Table S1. Integrals of the main peaks of the curing mixture at different temperatures; Figure S6. (a) Thermogravimetric curves in N_2_ atmosphere and (b) their derivatives for the materials prepared; Figure S7. Storage modulus of DIDE-PPO/MXDA and DIDE-PPO/IPDA as a function of angular frequency at different temperatures; Figure S8. Tension mode stress relaxation profiles of the prepared materials (colored lines) fit by a stretched exponential decay function (dashed black lines); Table S2. Fitting parameters for the stretched exponential decay function for the prepared materials; Figure S9. Storage and Loss Moduli of DIDE-PPO/MXDA as a function of the angular frequency; Figure S10. Storage and Loss Moduli of DIDE-PPO/IPDA as a function of the angular frequency; Figure S11. Evolution of storage modulus and tan δ with temperature for the prepared composite materials; Table S3. Main thermo-mechanical properties of the prepared composite materials; Table S4. Creep rate and strain recovery for the prepared carbon fiber reinforced composites.

## References

[1] Guerre M., Taplan C., Winne J.M., Du Prez F.E. (2020). Vitrimers: Directing chemical reactivity to control material properties. Chem. Sci..

[2] Lucherelli M.A., Duval A., Avérous L. (2022). Biobased vitrimers: Towards sustainable and adaptable performing polymer materials. Prog. Polym. Sci..

[3] Kloxin C.J., Bowman C.N. (2013). Covalent adaptable networks: Smart, reconfigurable and responsive network systems. Chem. Soc. Rev..

[4] Zheng J., Png Z.M., Ng S.H., Tham G.X., Ye E., Goh S.S., Loh X.J., Li Z. (2021). Vitrimers: Current research trends and their emerging applications. Mater. Today.

[5] Lessard J.J., Scheutz G.M., Sung S.H., Lantz K.A., Epps T.H., Sumerlin B.S. (2020). Block copolymer vitrimers. J. Am. Chem. Soc..

[6] Scheutz G.M., Lessard J.J., Sims M.B., Sumerlin B.S. (2019). Adaptable crosslinks in polymeric materials: Resolving the intersection of thermoplastics and thermosets. J. Am. Chem. Soc..

[7] Zhao X.-L., Tian P.-X., Li Y.-D., Zeng J.-B. (2022). Biobased covalent adaptable networks: Towards better sustainability of thermosets. Green Chem..

[8] Engelen S., Van Lijsebetten F., Aksakal R., Winne J.M., Du Prez F.E. (2023). Enhanced Viscosity Control in Thermosets Derived from Epoxy and Acrylate Monomers Based on Thermoreversible Aza-Michael Chemistry. Macromolecules.

[9] Montarnal D., Capelot M., Tournilhac F., Leibler L. (2011). Silica-Like Malleable Materials from Permanent Organic Networks. Science.

[10] Taynton P., Ni H., Zhu C., Yu K., Loob S., Jin Y., Qi H.J., Zhang W. (2016). Repairable Woven Carbon Fiber Composites with Full Recyclability Enabled by Malleable Polyimine Networks. Adv. Mat..

[11] Ciaccia M., Di Stefano S. (2015). Mechanisms of imine exchange reactions in organic solvents. Org. Biomol. Chem..

[12] Taynton P., Yu K., Shoemaker R.K., Jin Y., Qi H.J., Zhang W. (2014). Heat or water driven malleability in a highly recyclable covalent network polymer. Adv. Mater..

[13] Taynton P., Zhu C.P., Loob S., Shoemaker R., Pritchard J., Jin Y.H., Zhang W. (2016). Re-healable polyimine thermosets polymer composition and moisture sensitivity. Polym. Chem..

[14] Whiteley J.M., Taynton P., Zhang W., Lee S.H. (2015). Ultra-thin solid-state Li-ion electrolyte membrane facilitated by a self- healing polymer matrix. Adv. Mater..

[15] Liang K., Zhang G., Zhao J., Shi L., Cheng V., Zhang J. (2021). Malleable, recyclable, and robust poly(amide–imine) vitrimers prepared through a green polymerization process. ACS Sustain. Chem. Eng..

[16] Liu H., Zhang H., Wang H., Huang X., Huang G., Wu J. (2019). Weldable, malleable and programmable epoxy vitrimers with high mechanical properties and water insensitivity. Chem. Eng. J..

[17] Telatin T., De la Flor S., Serra À., Montané X. (2024). Poly(epoxy-imine) vitrimers. Effect of the structure on the stress relaxation and creep resistance. Polym. Test..

[18] Konuray A.O., Fernández-Francos X., Ramis X. (2017). Latent curing of epoxy-thiol thermosets. Polymer.

[19] Zhao S., Abu-Omar M.M. (2023). Recyclable and Malleable Epoxy Thermoset Bearing Aromatic Imine Bonds. Macromolecules.

[20] Miao P., Leng X., Liu J., Song G., He M., Li Y. (2022). Regulating the Dynamic Behaviors of Transcarbamoylation-Based Vitrimers via Mono-Variation in Density of Exchangeable Hydroxyl. Macromolecules.

[21] Roig A., Hidalgo P., Ramis X., De la Flor S., Serra À. (2022). Vitrimeric epoxy-amine polyimine networks based on a renewable vanillin derivative. ACS Appl. Polym. Mater..

[22] Zhao X.L., Liu Y.Y., Weng Y., Li Y.D., Zeng J.B. (2020). Sustainable epoxy vitrimers from epoxidized soybean oil and vanillin. ACS Sustain. Chem. Eng..

[23] Ding X.-M., Chen L., Xu Y.-J., Shi X.-H., Luo X., Song X., Wang Y.-Z. (2023). Robust Epoxy Vitrimer with Simultaneous Ultrahigh Impact Property, Fire Safety, and Multipath Recyclability via Rigid-Flexible Imine Networks. ACS Sustain. Chem. Eng..

[24] Yu Q., Peng X., Wang Y., Geng H., Xu A., Zhang X., Xu W., Ye D. (2019). Vanillin-based degradable epoxy vitrimers: Reprocessability and mechanical properties study. Eur. Polym. J..

[25] Roig A., Petrauskaitė A., Ramis X., De la Flor S., Serra À. (2022). Synthesis and characterization of new bio-based poly-(acylhydrazone) vanillin vitrimers. Polym. Chem..

[26] Verdugo P., Santiago D., De la Flor S., Serra À. (2024). A Biobased Epoxy Vitrimer with Dual Relaxation Mechanism: A Promising Material for Renewable, Reusable, and Recyclable Adhesives and Composites. ACS Sustain. Chem. Eng..

[27] Chen X., Li L., Wei T., Venerus D.C., Torkelson J.M. (2019). Reprocessable Polyhydroxyurethane Network Composites: Effect of Filler Surface Functionality on Cross-link Density Recovery and Stress Relaxation. ACS Appl. Mater. Inter..

[28] Meng F., Saed M.O., Terentjev E.M. (2019). Elasticity and Relaxation in Full and Partial Vitrimer Networks. Macromolecules.

[29] Van Lijsebetten F., Debsharma T., Winne J.M., Du Prez F.E. (2022). A Highly Dynamic Covalent Polymer Network without Creep: Mission Impossible?. Angew. Chem. Int..

[30] Yu S., Zhang G., Wu S., Tang Z., Guo B., Zhang L. (2020). Effects of dynamic covalent bond multiplicity on the performance of vitrimeric elastomers. J. Mater. Chem. A.

[31] Li L., Chen X., Jin K., Torkelson J.M. (2018). Vitrimers designed both to strongly suppress creep and to recover original cross-link density after reprocessing: Quantitative theory and experiments. Macromolecules.

[32] Porath L.E., Evans C.M. (2021). Importance of Broad Temperature Windows and Multiple Rheological Approaches for Probing Viscoelasticity and Entropic Elasticity in Vitrimers. Macromolecules.

[33] Yuan Y., Sun Y., Yan S., Zhao J., Liu S., Zhang M., Zheng X., Jia L. (2017). Multiply fully recyclable carbon fibre reinforced heat-resistant covalent thermosetting advanced composites. Nat. Commun..

[34] Liu Y., Lu F., Xu N., Wang B., Yang L., Huang Y., Hu Z. (2022). Mechanically robust, hydrothermal aging resistant, imine-containing epoxy thermoset for recyclable carbon fiber reinforced composites. Mater. Des..

[35] Shaw M.T., MacKnight W.J. (2005). Introduction to Polymer Viscoelasticity.

[36] Sperling L.H. (2005). Introduction to Physical Polymer Science.

[37] Kelton K.F. (2017). Kinetic and structural fragility—A correlation between structures and dynamics in metallic liquids and glasses. J. Phys. Condens. Matter.

[38] Appelt B.K., Cook P.J., Gill P.S., Johnson Julian F. (1984). Defining B-stage and total cure for a dicy based epoxy resin by DSC. Analytical Calorimetry Volume 5.

[39] Ruiz De Luzuriaga A., Martin R., Markaide N., Rekondo A., Cabañero G., Rodríguez J. (2016). Epoxy resin with exchangeable disulfide crosslinks to obtain reprocessable, repairable and recyclable fiber-reinforced thermoset composites. Mater. Horiz..

[40] Grunenfelder L.K., Nutt S.R. (2012). Prepreg age monitoring via differential scanning calorimetry. J. Reinf. Plast. Compos..

