# Supplementary material for: Chemically Degradable Vitrimers Based on Divanillin Imine Diepoxy Monomer and Aliphatic Diamines for Enhanced Carbon Fiber Composite Applications
Source: Polymers (Basel). 2024 Sep 29;16(19):2754. doi: 10.3390/polym16192754 (PMC11479291; doi:10.3390/polym16192754)
Supplement: Supplementary file 1 [file polymers-16-02754-s001.zip › polymers-3217087-supplementary.pdf]

Supplementary Material

Chemically Degradable Vitrimers Based on Divanillin Imine Diepoxy Monomer and Aliphatic Diamines for Enhanced Carbon Fiber Composite Applications

Tommaso Telatin [1], Silvia De la Flor [2], Xavier Montané [1,*] and Àngels Serra [1,*]

1 Department of Analytical and Organic Chemistry, Universitat Rovira i Virgili, C/Marcel·lí Domingo 1, 43007 Tarragona, Spain
2 Department of Mechanical Engineering, Universitat Rovira i Virgili, Av. Països Catalans 26, 43007 Tarragona, Spain
* Correspondence: xavier.montane@urv.cat (X.M.); angels.serra@urv.cat (À.S.); Tel.: +34-977559768 (X.M.); +34-977559768 (À.S.)

Table of Contents:

1. Structural characterization of the monomer S3

2. Study of the curing process by ^{1}H NMR spectroscopy S5

3. Thermogravimetric analysis of the prepared materials S6

4. Storage moduli as a function of the angular frequency for the prepared materials S7

5. Stress relaxation profiles and Kohlrausch-Williams-Watts (KKW) model analysis S8

6. Frequency sweeps experiments for DIDE-PPO/MXDA S9

7. Frequency sweeps experiments for DIDE-PPO/IPDA S10

8. Carbon fibers reinforced composites S11

1. Structural characterization of the monomer

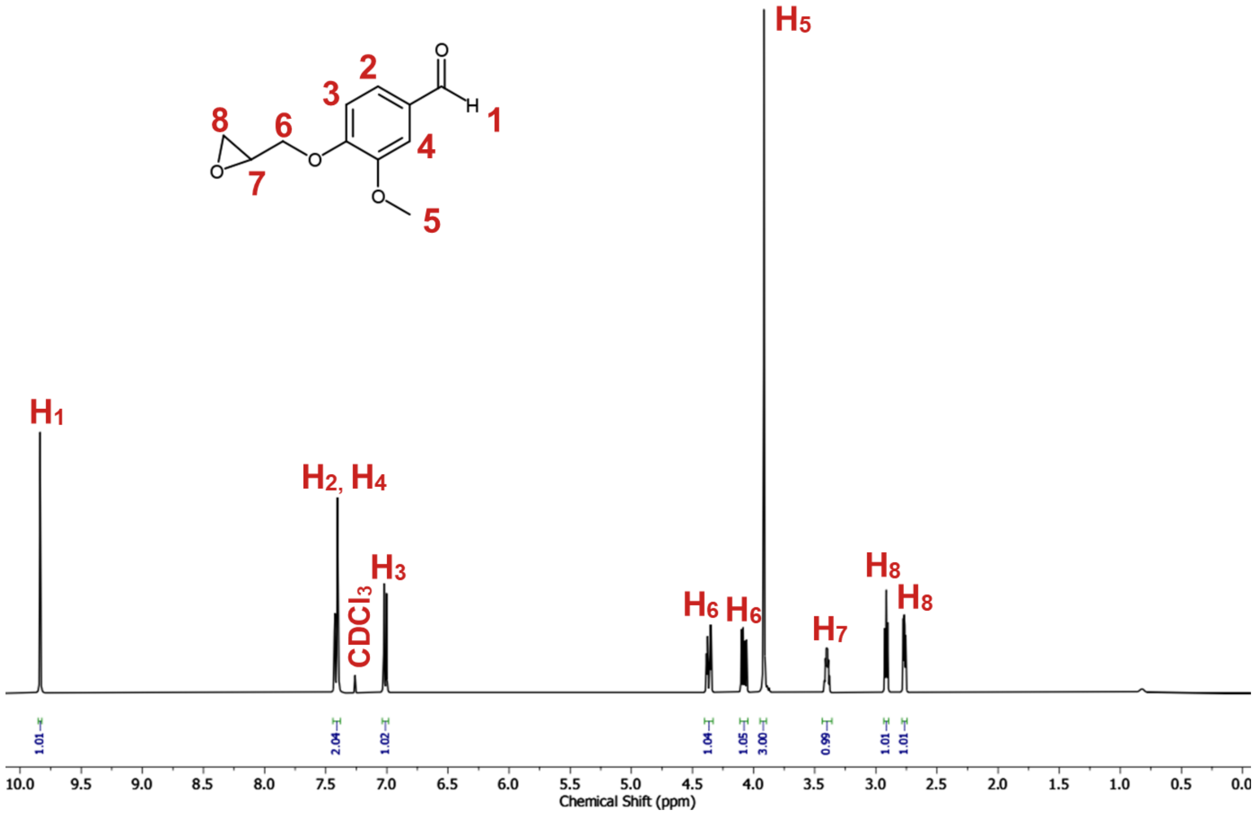

Figure S1. ^{1}H NMR spectrum of EVan in $CDCl_3$.

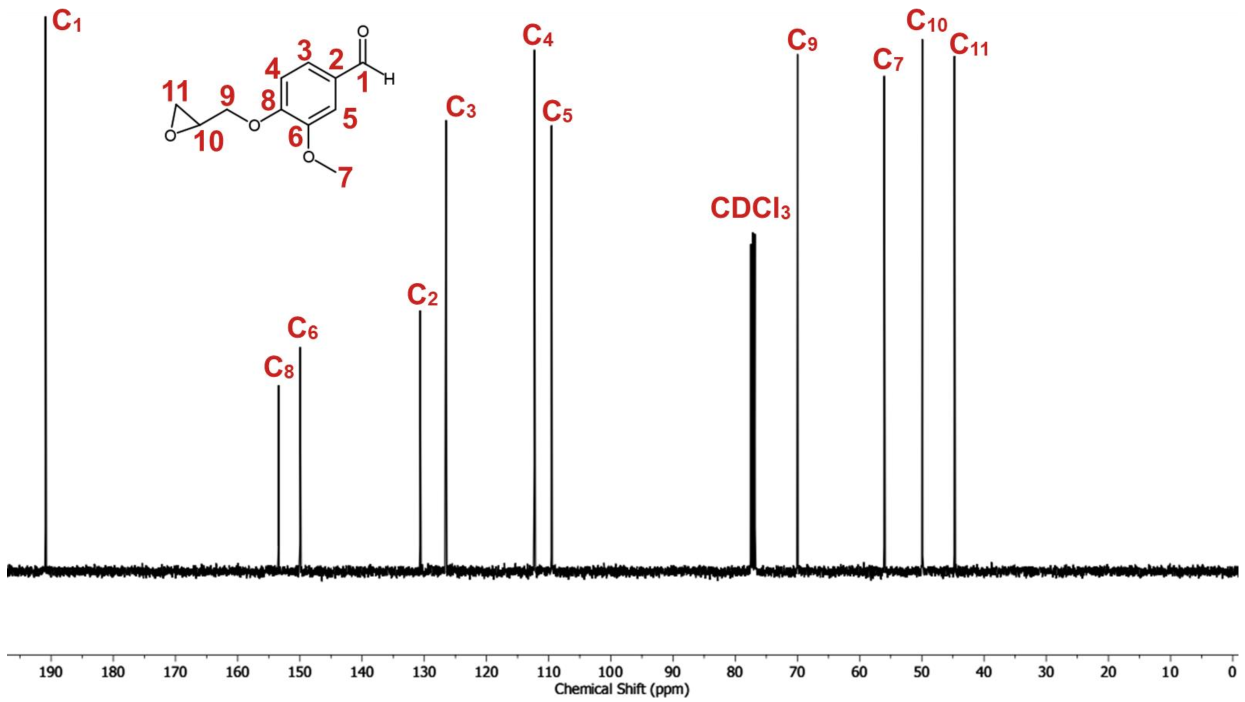

Figure S2. ^{13}C NMR spectrum of EVan in $CDCl_3$.

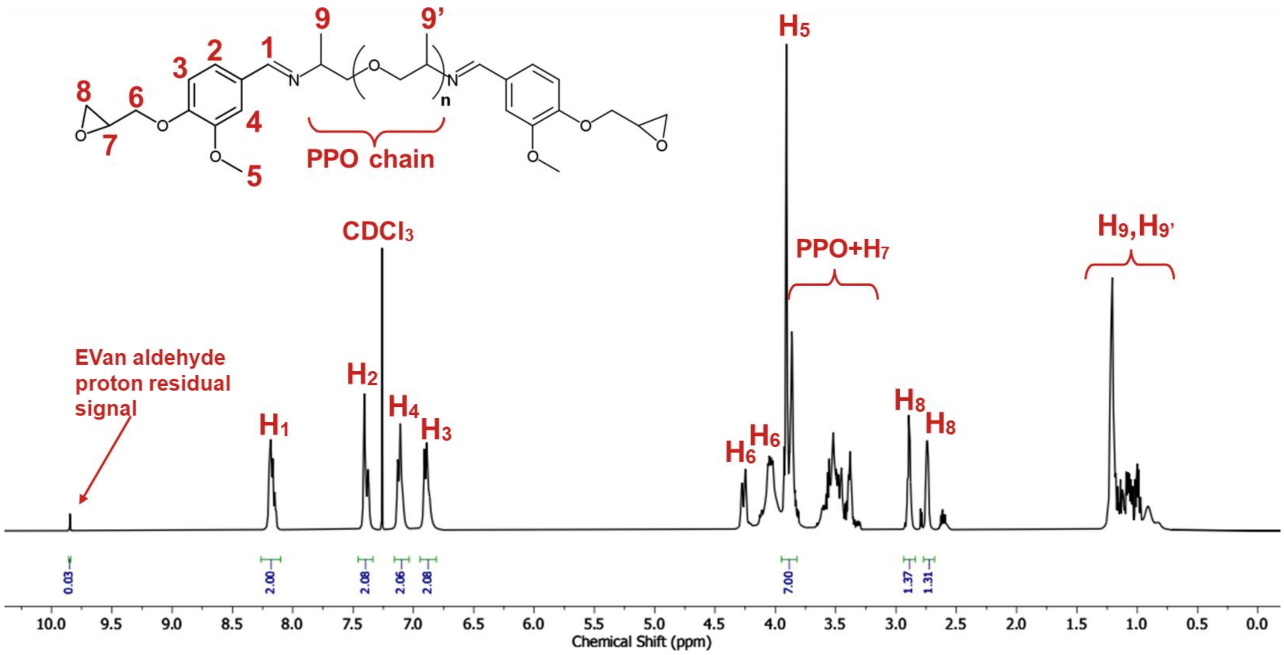

Figure S3. ^{1}H NMR spectrum of DIDE-PPO in $CDCl_3$.

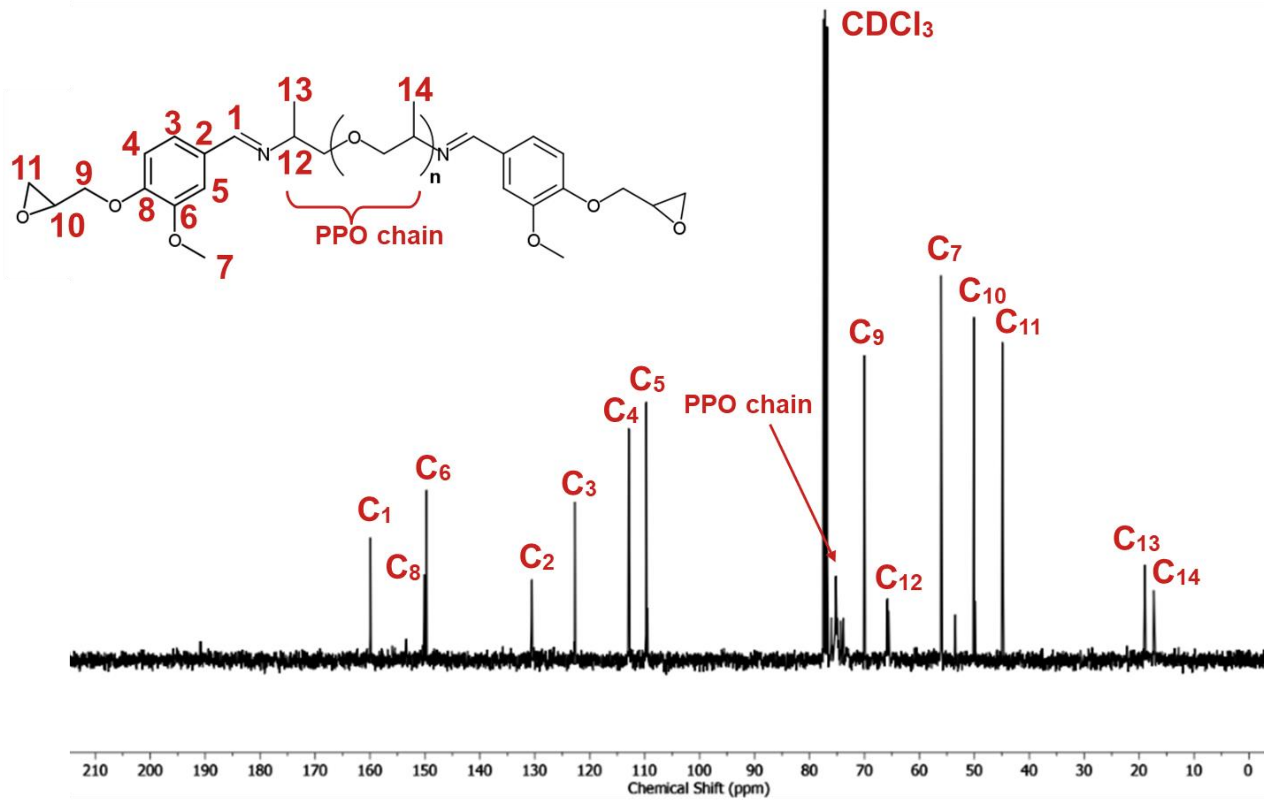

Figure S4. ^{13}C NMR spectrum of DIDE-PPO in $CDCl_3$.

2. Study of the curing process by ^{1}H NMR spectroscopy

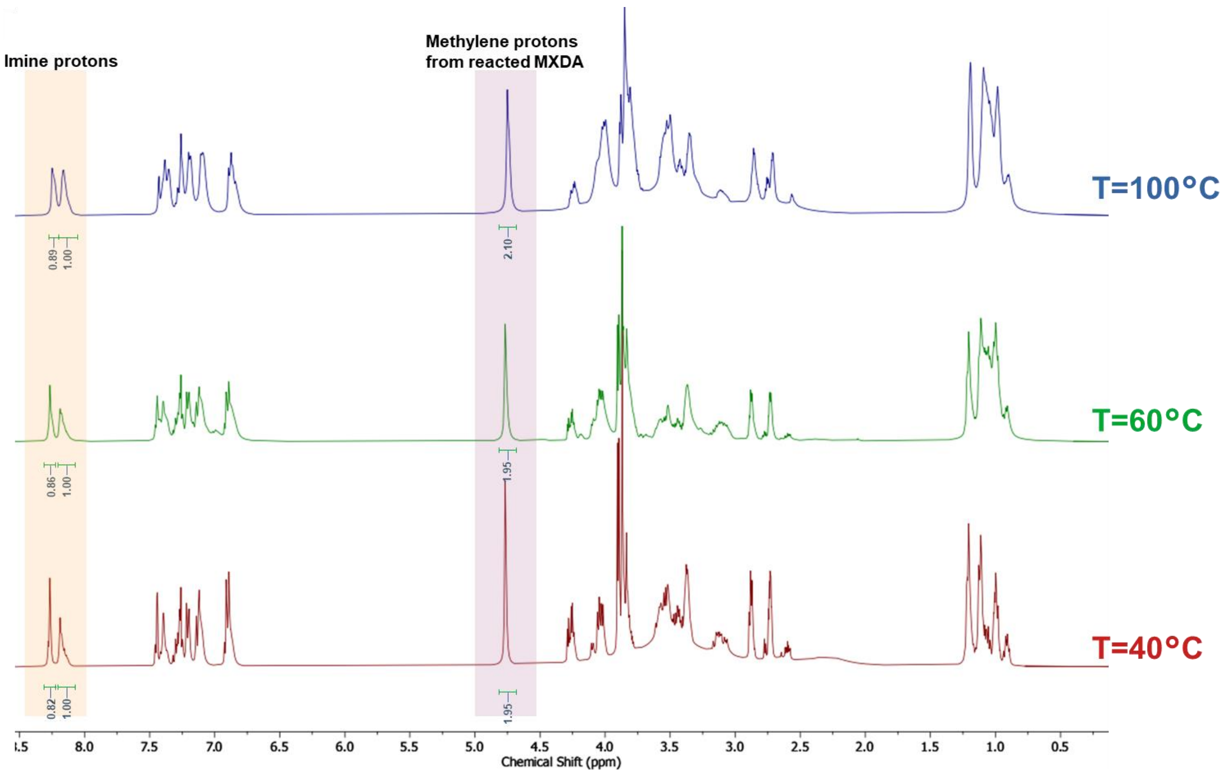

Figure S5. ^{1}H NMR spectra in $CDCl_3$ of the curing mixture at 40, 60 and 100 °C collected after 10 minutes of equilibration at each temperature.

Table S1. Integrals of the main peaks of the curing mixture at different temperatures.

Temperature (°C)	$I_{8.27\ ppm}$[a]	$I_{8.18\ ppm}$[b]	$I_{4.77\ ppm}$[c]	$I_{8.18\ ppm}/I_{8.27\ ppm}$[d]
40	1.00	0.82	1.95	0.82
60	1.00	0.86	1.95	0.86
100	1.00	0.89	2.10	0.89

[a] Normalized integral of the original imine proton signal.

[b] Normalized integral of the MXDA-derived imine proton signal.

[c] Normalized integral of the methylene protons signal relative to the reacted MXDA hardener.

[d] Ratio between the integrals of the two imine signals.

3. Thermogravimetric analysis of the prepared materials

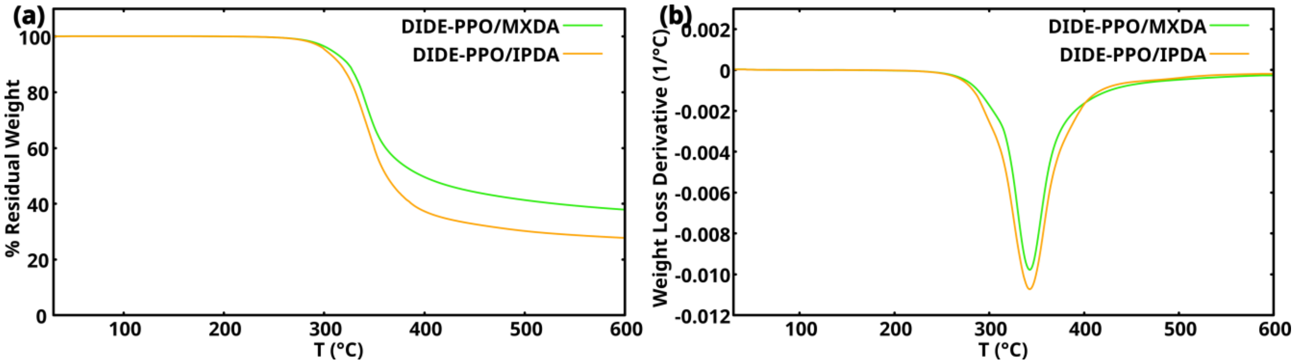

Figure S6. (a) Thermogravimetric curves in N_2 atmosphere and (b) their derivatives for the materials prepared.

4. Storage Moduli as a function of the angular frequency for the prepared materials

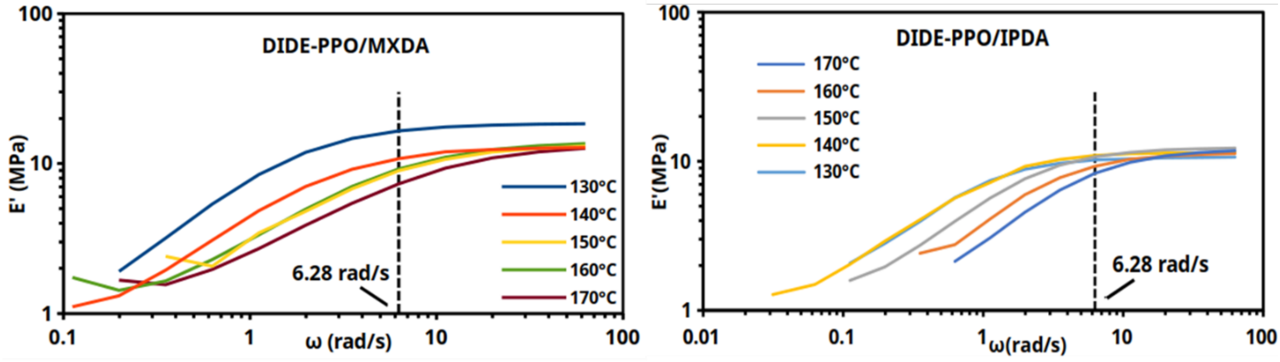

Figure S7. Storage modulus of DIDE-PPO/MXDA and DIDE-PPO/IPDA as a function of angular frequency at different temperatures.

5. Stress relaxation profiles and Kohlrausch-Williams-Watts (KKW) model analysis

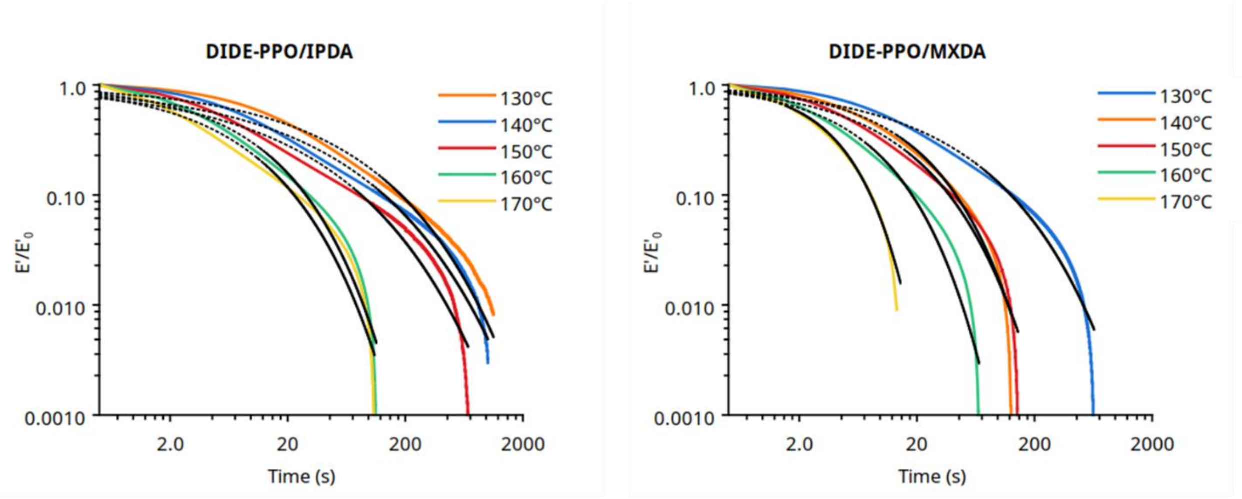

Figure S8. Tension mode stress relaxation profiles of the prepared materials (colored lines) fit by a stretched exponential decay function (dashed black lines).

Table S2. Fitting parameters for the stretched exponential decay function for the prepared materials.

Sample	T (°C)	β^a	τ^b (s)	R^2
	130	0.46	29.64	0.976
	140	0.41	17.39	0.962
**DIDE-PPO/IPDA**	150	0.41	11.27	0.958
	160	0.46	7.36	0.976
	170	0.57	5.22	0.955
	130	0.49	22.95	0.981
	140	0.68	12.49	0.991
**DIDE-PPO/MXDA**	150	0.60	9.38	0.980
	160	0.71	5.55	0.976
	170	1.00	3.42	0.983

[a] Stretched exponential parameter.

[b] Characteristic relaxation time.

6. Frequency sweeps experiments for DIDE-PPO/MXDA

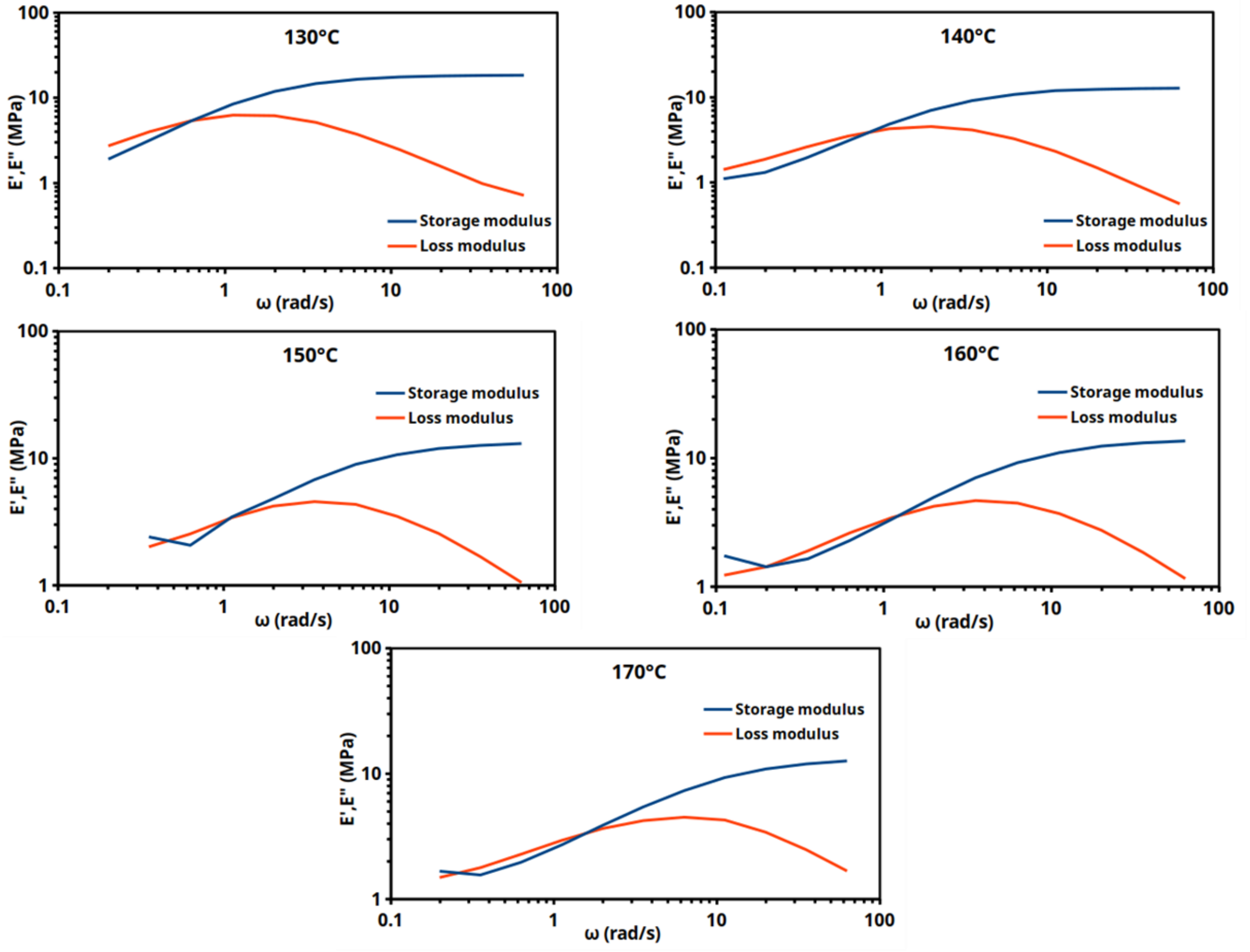

Figure S9. Storage and Loss Moduli of DIDE-PPO/MXDA as a function of the angular frequency.

7. Frequency sweeps experiments for DIDE-PPO/IPDA

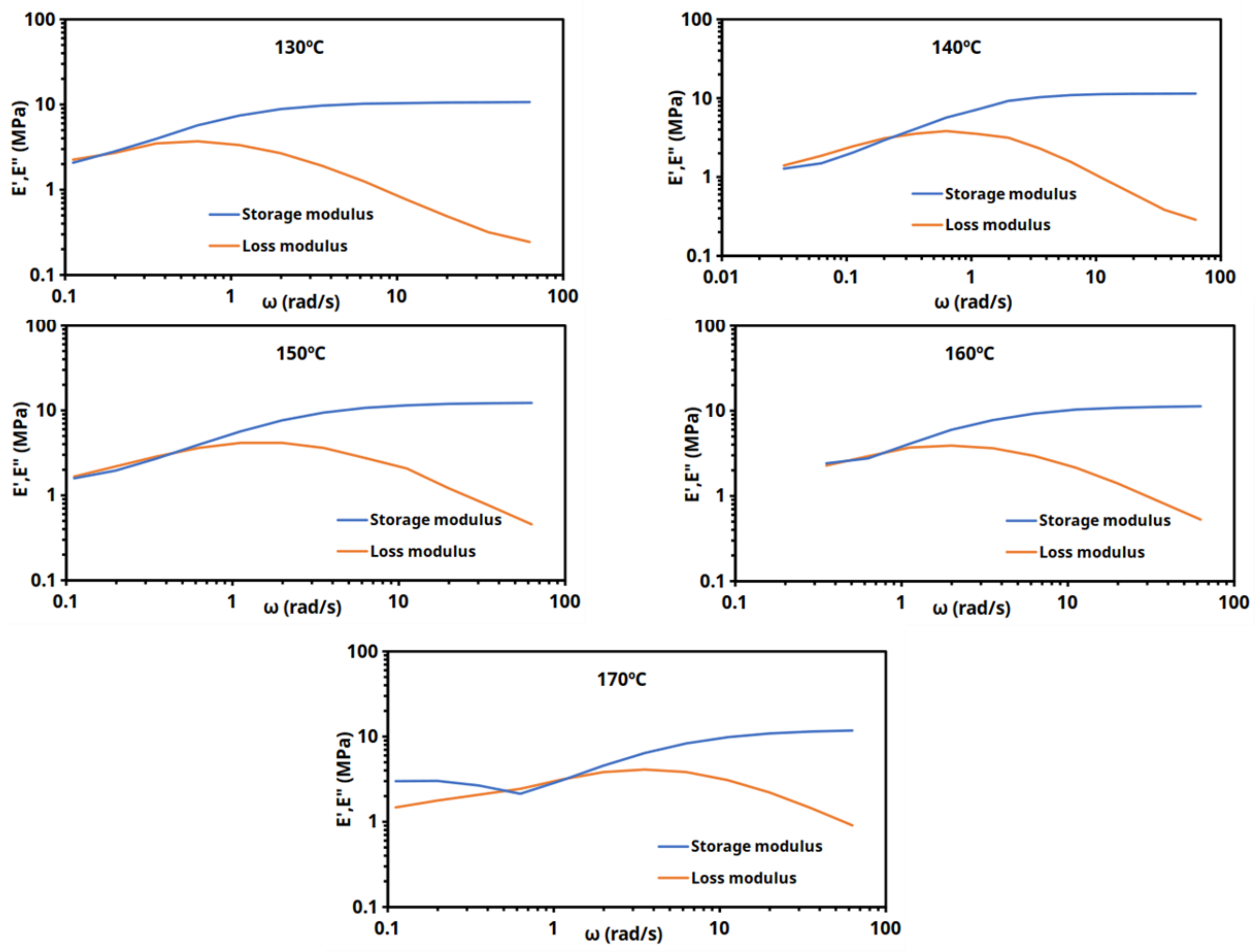

Figure S10. Storage and Loss Moduli of DIDE-PPO/IPDA as a function of the angular frequency.

8. Carbon fibers reinforced composites

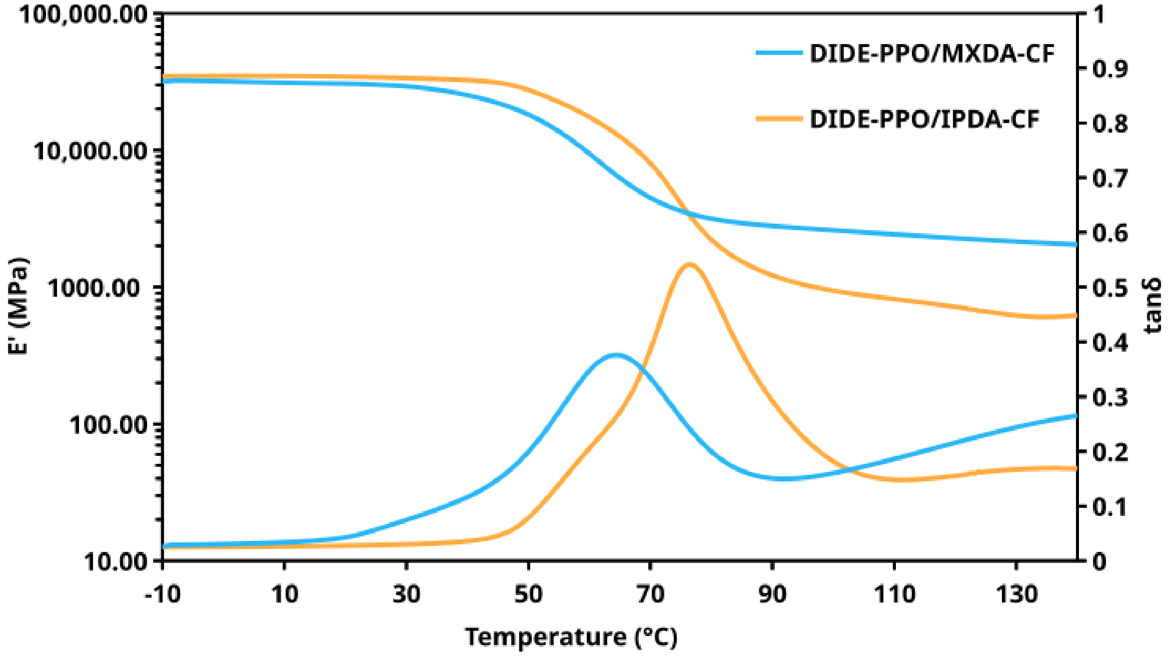

Figure S11. Evolution of storage modulus and tan δ with temperature for the prepared composite materials.

Table S3. Main thermomechanical properties of the prepared composite materials.

Sample	E'_{Glassy} [a] (MPa)	$E'_{Rubbery}$ [b] (MPa)	$T_{\tan\delta}$ [c]
**DIDE-PPO/IPDA-CF**	34422	708	76
**DIDE-PPO/MXDA-CF**	31146	2427	64

Table S4. Creep rate and strain recovery for the prepared carbon fiber reinforced composites.

Sample	dε/dt [a]	% Residual deformation [b]
**DIDE-PPO/IPDA**	2.39×10^{-3}	0.61
**DIDE-PPO/MXDA**	2.13×10^{-3}	0.37
**DIDE-PPO/IPDA-CF**	4.24×10^{-4}	0.13
**DIDE-PPO/MXDA-CF**	4.12×10^{-4}	0.02

[a] Determined from the slope of the steady-state region of the creep curve.

[b] Determined after 30 min of releasing the stress.
